# *Neoprotoparmelia* gen. nov. and *Maronina* (Lecanorales, Protoparmelioideae): species description and generic delimitation using DNA barcodes and phenotypical characters

**DOI:** 10.3897/mycokeys.44.29904

**Published:** 2018-12-14

**Authors:** Garima Singh, André ptroot, Víctor J. ico, Jürgen tte, Pradeep K. Divakar, Ana Crespo, Marcela Eugenia da Silva Cáceres, H. Thorsten Lumbsch, Imke Schmitt

**Affiliations:** 1 Senckenberg Biodiversity and Climate Research Centre (SBiK-F), Senckenberganlage 25, 60325, Frankfurt am Main, Germany; 2 Laboratório de Botânica/Liquenologia, Centro de Ciências Biológicas e da Saúde, Universidade Federal de Mato Grosso do Sul, Caixa Postal 549, CEP 79070-900, Campo Grande, Mato Grosso do Sul, Brazil; 3 Departamento de Farmacología, Farmacognosia y Botánica, U.D. Botánica, Facultad de Farmacia, Universidad Complutense, Plaza de Ramón y Cajal s/n, E-28040 Madrid, Spain; 4 Universidade Federal de Sergipe, Departamento de Biociências, Itabaiana, Sergipe, Brazil; 5 Science & Education, Field Museum of Natural History, 1400 S. Lake Shore Drive, Chicago, IL, 60605, United States of America; 6 Department of Biological Sciences, Max-von-Laue-Str. 9, 60438 Frankfurt am Main, Germany

**Keywords:** ITS, lichenised fungi, BPP, new genus, new species, Parmeliaceae, taxonomy

## Abstract

Multilocus phylogenetic studies revealed a high level of cryptic diversity within the lichen-forming fungal genus *Maronina* (Protoparmelioideae, Parmeliaceae). Coalescent-based species delimitation suggested that most of the cryptic molecular lineages warranted recognition as separate species. Here we study the morphology and chemistry of these taxa and formally describe eight new species based on phenotypical and molecular characters. Further, we evaluate the use of ITS rDNA as a DNA barcode for identifying species in this genus. For the first time, we obtained an ITS sequence of *Maroninaaustraliensis*, the type species of the genus and showed that it is phylogenetically not closely related to species currently placed in *Maronina* or *Protoparmelia*. We assembled a dataset of 66 ITS sequences to assess the interspecies genetic distances amongst the twelve *Maronina* species using ITS as DNA barcode. We found that *Maronina* and *Protoparmelia* form a supported monophyletic group whereas *M.australiensis* is sister to both. We therefore propose a new genus *Neoprotoparmelia* to accommodate the tropical-subtropical species within Protoparmelioideae, with *Neoprotoparmeliacorallifera* as the type, *N.amerisidiata*, *N.australisidiata*, *N.brasilisidiata*, *N.capensis*, *N.crassa*, *N.pauli*, *N.plurisporibadia* and *N.siamisidiata* as new species and *N.capitata*, *N.isidiata*, *N.multifera*, *N.orientalis* and *N.pulchra* as new proposed combinations. We provide a key to *Neoprotoparmelia* and confirm the use of ITS for accurately identifying species in this group.

## Introduction

The taxonomic status of the genus *Maronina* and its phylogenetic relationships have been a matter of debate. *Maronina* was formally described in 1990 (Hafellner and Rogers 1990) for two species with multispored asci, the type species *Maroninaaustraliensis* and *M.multifera*. The authors suggested a close relationship of *Maronina* and *Protoparmelia* based on ascus characters and considered *Maronina* a multispored derivative of *Protoparmelia*. Later, molecular data confirmed the phylogenetic relationship between *Maronina* with *Protoparmelia* and *Maronina* was merged with *Protoparmelia* (Papong et al. 2011). Recently, Kraichak et al. (2017) suggested the use of a temporal banding approach for a consistent grouping of taxa at higher taxonomic levels, i.e. at family and genus level, for lichen-forming fungi. This approach identifies a divergence time of ~102–112 Ma for families and 29–33 Ma for genera (Kraichak et al. 2017). Based on this approach, the genus *Maronina* has been resurrected (*Maronina*-*Protoparmelia* split ~70 Ma; Divakar et al. 2017, Kraichak et al. 2017, Singh et al. 2018) and both genera, *Protoparmelia* and *Maronina*, have been placed together in the subfamily Protoparmelioideae (Parmeliaceae). Currently, the genus *Protoparmelia* comprises arctic, boreal, temperate and Mediterranean species, whereas the genus *Maronina* comprises subtropical and tropical species.

Presently *Maronina* includes 11 species (Aptroot 2002, Aptroot et al. 1997a, 1997b, 2007, 2013, Barbero et al. 2006, Elix 2007, 2009, Hafellner and Rogers 1990, Kantvilas and Elix 2007, 2010, Lendemer and Lumbsch 2008, Papong et al. 2011). Molecular data are available for six species (*Maroninacapitata*, *M.corallifera*, *M.multifera*, *M.orientalis*, *M.isidiata* and *M.pulchra*). A recent study aimed at molecular identification of species in *Maronina* and *Protoparmelia*, based on a multilocus dataset and species delimitation analysis, included these six species and two putatively novel species (Singh et al. 2015). Molecular analysis confirmed the presence of the above-mentioned species in *Maronina* and suggested seven additional species: *M.isidiata* A, *M.isidiata* B (Brazil), *M.isidiata* C (Thailand), *M.isidiata* D (Australia), *M.isidiata* E (Australia), *M.* ZA (South Africa) and *M.* KE (Kenya). These candidate species were strongly supported by species delimitation approaches BP&P and speDeSTEM (Singh et al. 2015), but not formally proposed at the time. In the present study, we describe the seven novel *Maronina* species *sensu*Singh et al. (2015) and a further new saxicolous species from Brazil based on phenotypical and molecular evidence. In addition, we include the type specimen *M.australiensis*, which has not been sequenced before.

## Materials and methods

We included 66 ITS rDNA sequences of *Protoparmelia* and *Maronina* in this study. Out of these, 61 ITS sequences are from Singh et al. (2015) and five sequences are new, representing two additional specimens of *M.capitata*, two sequences of a new taxon *M.plurisporibadia* and a sequence of the type species of the genus *Maronina*, *M.australiensis* (Table 1).

### Molecular methods

For DNA extraction, amplification and sequencing, we followed the protocols from Singh et al. (2015). We used the *Protoparmelia* specific primers (Suppl. material 1:Table S1) and Ex Taq polymerase (Takara Bio Europe, France) for the PCRs. Generating an ITS sequence from the 32-year-old *M.australiensis* sample required a PCR cloning approach. The amplified products were cloned into the pJET1.2 / blunt cloning vector using the Thermo Scientific CloneJET PCR cloning kit and transformed into *E.coli* XL1-Blue cells (for details see: https://www.chem-agilent.com/pdf/strata/200249.pdf). The cloned PCR products were analysed using the “colony PCR”. For the PCR reactions and sequencing, we used the pJET1.2 Forward Sequencing Primer and the pJET1.2 Reverse Sequencing Primer. We performed a BLAST search using the *M.australiensis* ITS sequence to infer the phylogenetic affinities of *M.australiensis*.

### Phylogenetic analyses

**Figure 1.**
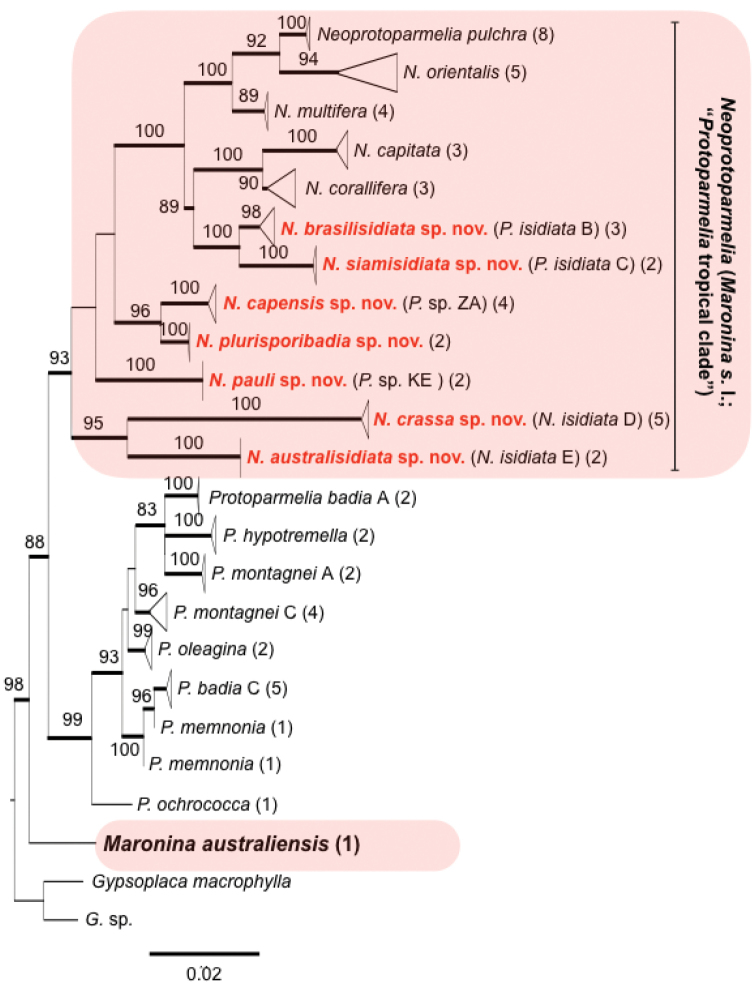
Phylogeny of Protoparmelioideae based on maximum likelihood analysis of ITS rDNA sequences of *Protoparmelia* and *Neoprotoparmelia* (*Maronina* s.l.). Numbers above branches indicate bootstrap support. Terminal clades were collapsed at the species level for clarity of presentation. The length of the triangle corresponds to branch lengths. Numbers in parentheses indicate number of specimens included in collapsed clade. Voucher information of each specimen in a clade is given in Table 1; New *Neoprotoparmelia* species are marked in red and bold.

We aligned the sequences using MAFFT v5 with Geneious version 5.6.5 (Katoh et al. 2005, Drummond et al. 2011). To infer the phylogenetic position of *M.australiensis* within Protoparmelioideae, we produced an alignment using the ITS sequences of *M.australiensis*, *Protoparmelia* and *Maronina* species. Using this alignment, we generated a maximum likelihood tree using the ITS sequences from *Protoparmelia* (9 species, 20 sequences) and *Maronina* (13 species including the type species, 44 sequences; Fig. 1), with GTR + G as the substitution model. This dataset contains overall 66 sequences, including 2 sequences of the outgroup (Gypsoplacaceae). The maximum likelihood search was performed using the RAxML-HPC BlackBox v8.1.11 on the Cipres Scientific gateway (Miller et al. 2010, Stamatakis 2014).

**Table 1.** Specimens used in this study. New sequences are indicated in bold.

Species	Sample ID as in BOLD database	Voucher	Accession number ITS rDNA
* Gypsoplaca macrophylla *	NA	USA, Rosentreter 15995 (F)	KF650781
*Gypsoplaca* sp.	NA	USA, Spribille 38752 (GZU)	MK046745
* Maronina australiensis *	NA	Australia, Hafellner 17823 & Rogers, holotype (GZU)	MK046744
* Neoprotoparmelia australisidiata *	IS120074	Australia, Kantvilas 228/10, HO 559228 (HO)	KP822275
	IS120075	Australia, Kantvilas 289/07, HO 545660 (HO)	KP822276
* N. brasilisidiata *	IS140153	Brazil, Cáceres & Aptroot ISE 21684, holotype (ISE)	KP822271
	IS140154	Brazil, Cáceres & Aptroot ISE 13673 (ABL)	KP822272
	IS140192	Brazil, Cáceres & Aptroot 21648 (ISE)	KY066262
* N. capensis *	ZA120814	South Africa, Crespo, Divakar, Hawksworth, Amo & Lumbsch 14c, MAF-Lich. 19627, isotype (MAF)	KP822302
	ZA120815	South Africa, Crespo, Divakar, Hawksworth, Amo & Lumbsch 39a, MAF-Lich. 19625, isotype (MAF)	KP822303
	ZA120816	South Africa, Crespo, Divakar, Hawksworth, Amo & Lumbsch 44e, MAF-Lich. 19628 isotype, (MAF)	KP822304
	ZA120817	South Africa, Crespo, Divakar, Hawksworth, Amo & Lumbsch 63f, MAF-Lich. 19584 holotype (MAF)	KY066279
* N. capitata *	CAJF821184	USA, Lendemer 9044 (NY)	JF821184
	CA140194	Brazil, Cáceres & Aptroot ISE 22138 (ISE)	MK046746
	CA140195	Brazil, Cáceres & Aptroot ISE 22207 (ISE)	MK046747
* N. corallifera *	CO120073	Thailand, Papong & Konhin 6601pp, 554585 (HO)	KY066260
	CO120744	Thailand, Papong 7100 (MSUT)	KY066261
	CO120302	Thailand, Papong 6483 (MSUT)	KP822264
* N. crassa *	IS120052	Australia, Elix 38202, CANB 800762 (CANB)	KY066265
	IS120053	Australia, Elix 38207, CANB 800763 (CANB)	KY066266
	IS120056	Australia, Elix 39795, CANB 783253 (CANB)	KP822273
	IS120057	Australia, Elix 39804, CANB 783259 (CANB)	KY066264
	IS120058	Australia, Elix 39805, CANB 783260 holotype (CANB)	KP822274
* N. multifera *	MU140152	Brazil, Cáceres & Aptroot ISE 13667 (ABL)	KP822291
	MU140152	Brazil, Cáceres & Aptroot Ise 13667 (ABL)	KP822292
	MU140198	Brazil, Cáceres & Aptroot, ISE 9559 (ISE)	KY066270
	MU140201	Brazil, Cáceres & Aptroot ISE 22119 (ISE)	KY066271
* N. orientalis *	OR120077	Thailand, Papong 6612, HO-554582 (HO)	KY066274
	OR120296	Thailand, Papong 6922 (MSUT)	KP822295
	OR120298	Thailand, Papong 7033 (MSUT)	KP822296
	OR120301	Thailand, Papong 6487 (MSUT)	KP822297
	ORJF821182	Thailand, Papong 6922 (MSUT)	JF821182
* N. pauli *	Ke1	Kenya, Kirika & Lumbsch 3821-1 holotype (EA)	KP822279
	Ke2	Kenya, Kirika & Lumbsch 3821-2 isotype (F)	KP822280
* N. plurisporibadia *	140189	Brazil, Cáceres & Aptroot ISE 22130 holotype (ABL)	MK046748
	140190	Brazil, Cáceres & Aptroot ISE 22161 (ABL)	MK046749
* N. pulchra *	PU120061	Australia, Elix 37379, CANB 803643 (CANB)	KY066277
	PU120062	Australia, Elix 38452, CANB 769060, (CANB)	KY066276
	PU120063	Australia, Elix 39560, CANB 789446 (CANB)	KP822298
	PU120064	Australia, Elix 37097, CANB 800711 (CANB)	KP822299
	PU120066	Australia, Elix 39787, CANB 781897 (CANB)	KP822300
	PU120067	Australia, Elix 39791, CANB 783250 (CANB)	KY066275
	PU120068	Australia, Elix 39798, CANB 783256 (CANB)	KY066278
	PU120069	Australia, Elix 39806, CANB 783261 (CANB)	KP822301
* N. siamisidiata *	130029	Thailand, P. & B. v.d. Boom 46872 (Hb. v.d. Boom)	KP822277
	130030	Thailand, P. & B. v.d. Boom 46947 (Hb. v. d. Boom)	KP822278
*Protoparmeliabadia* A	NA	Austria, Muggia & Hafellner 68478 (GZU)	KF562191
*P.badia* A	NA	Slovenia, Hafellner 71474 (GZU)	KP822209
*P.badia* B1	NA	Italy, Dal Grande & Singh FR 68881 (FR)	KP822251
	NA	Italy, Dal Grande & Singh FR 68882 (FR)	KP822252
	NA	Spain, v. d. Boom 46079 (Hb. v. d. Boom)	KP822242
*P.badia* C	NA	Spain, Crespo, Rico, Ruibal & Boluda, MAF-Lich. 19437 (MAF)	KP822260
	NA	Spain, Crespo, Rico, Ruibal & Boluda, MAF-Lich. 19438 (MAF)	KP822261
* P. hypotremella *	NA	Canada, Lendemer 14431B (NY)	KP822268
	NA	Canada, Lendemer 14563 (NY)	KP822269
* P. memnonia *	NA	Norway, Haugan 9612 (O)	KF562194
	NA	Norway, Holien 13370 (TRH)	KP822282
*P.montagnei* A	NA	Turkey, Crespo, Divakar, Lumbsch & Candan, MAF-Lich. 19465 (MAF)	KP822283
	NA	Turkey, Crespo, Divakar, Lumbsch & Candan, MAF-Lich. 19469 (MAF)	KP822286
*P.montagnei* C	NA	Spain, Crespo, Rico & Ruibal MAF-Lich. 19427 (MAF)	KP822288
	NA	Spain, Crespo, Rico & Ruibal MAF-Lich. 19428 (MAF)	KP822289
	NA	Spain, Crespo, Cubas, Núñez & Divakar, MAF-Lich. 19462 (MAF)	KY066267
	NA	Turkey, Divakar, Crespo, Candan & Lumbsch, MAF-Lich. 19467, (MAF)	KP822287
* P. ochrococca *	NA	USA, McCune 31673 (OSU)	KP822293
* P. oleagina *	NA	Norway, Johnsen, L-92691 (BG)	KY066273
	NA	Norway, Tønsberg 41328, L-92554 (BG)	KY066272

### Analysis of sequence variation in the ITS barcode marker

To infer intra- and interspecific ITS sequence variation within and amongst putative lineages of *Neoprotoparmelia* (*Maronina* s.l.), we calculated pairwise distances amongst *Neoprotoparmelia* species (*Maronina* s.l. species, 43 sequences from 12 species, excluding *M.australiensis* and the outgroup). Pairwise distances between different haplotypes were reported as the number of nucleotide substitutions per site (s/s). Average genetic distance was calculated on the BOLD workbench (Barcode of Life Data Systems, BOLD; Ratnasingham and Hebert 2007). The ITS distance was inferred based on pairwise comparisons of all sequences. ITS sequences from the candidate species circumscribed in Singh et al. (2015) and the newly generated sequences, including the voucher information, were submitted to the BOLD database, under the project name ‘*Neoprotoparmelia* species description’.

### Morphological and chemical methods

For the samples *Maroninaisidiata* A, *M.isidiata* B, *M.isidiata* C, *M.isidiata* D, *M.isidiata* E and *M.plurisporibadia* (in Singh et al. 2015), morphological examination was performed with an Olympus SZX7 and pictures were taken with Nikon Coolpix 995. Hand-made sections of ascomata and thallus were studied in water, 5% KOH (K) and/or Lugol’s reagent (1% I_2_) after pre-treatment with KOH (IKI). Microscopic photographs were prepared using an Olympus BX50 with Nomarski interference contrast and Nikon Coolpix 995.

For the samples *Maronina* ZA and *M.* KE, morphological examination was performed under a Nikon SMZ-1500 stereomicroscope and Nikon Eclipse-80i microscope, with bright field and DIC. Photographs were taken with a Nikon DS-Ri2 coupled to the microscope and stereomicroscope. Observations and measurements of ascospores and conidia were made in water. When possible, for each species, at least 30 spores and conidia from different specimens were measured and length width (l:b) were calculated. In the description of the new species, n (number of spores and conidia measured) are given in parentheses. Spot tests (K, C, I and Pd) and thin-layer chromatography (TLC) were carried out following Orange et al. (2010). We used TLC solvent system C (200 ml toluene / 30 ml acetic acid), with concentrated acetone extracts at 50 °C spotted on to silica gel 60 F254 aluminium sheets (Merck, Darmstadt).

## Results and discussion

In the ML phylogenetic tree of Protoparmelioideae, both *Protoparmelia* and *Maronina* s.l. form supported monophyletic clades (Fig. 1). *Protoparmelia* and *Maronina* s.l. are supported as sister groups, whereas *Maroninaaustraliensis* is sister to the *Protoparmelia*-*Maronina* s.l. clade. This suggests that *Maronina*, as currently circumscribed, is polyphyletic. The heterogeneous nature of *Maronina* has already been indicated by Kantvilas and Elix (2007), based on ascomatal characters. Originally, Hafellner and Rogers (1990) described two species in *Maronina*, namely *M.australiensis* from Australia and *M.multifera* from South America. Later, Kantvilas and Elix (2007) described another species, *M.hesperia*, from Australia and pointed out that *M.multifera* differs chemically and morphologically from *M.australiensis*. *Maroninaaustraliensis* and *M.hesperia* contain depsides instead of depsidones as found in *M.multifera* and paraphyses in *M.australiensis* and *M.hesperia* are slender and mostly simple, whereas those in *M.multifera* are branched and anastomosing. The authors suggested *Maronina* s.str. to be a strictly Australian genus, comprising *M.australiensis* and *M.hesperia* only. In the present study, we support this hypothesis, based on molecular evidence, which confirms that *M.australiensis* and *M.multifera* are not closely related. Instead, the morphological and chemical properties of *M.multifera* are very similar to the other *Maronina* s.l. species, e.g. presence of depsidones and branched paraphyses. *Maroninamultifera* forms a well-supported monophyletic clade with other *Maronina*. s.l. taxa (Fig. 1 this study and Singh et al. 2015, 2017 based on a 6-locus phylogeny). Based on molecular and phenotypical evidence, we thus propose to restrict the genus *Maronina* s.str. to *M.australiensis*, the type species of the genus and *M.hesperia*. In its restricted circumscription, the genus *Maronina* is currently only known from Australia. To accommodate the *Maronina* s.l. taxa, sister of *Protoparmelia*, we propose the new genus *Neoprotoparmelia* with *N.corallifera*, as the type species. The following species are here recognised in *Neoprotoparmelia*: *N.capitata*, *N.isidiata*, *N.multifera*, *N.orientalis* and *N.pulchra* and eight new described species as *N.amerisidiata*, *N.australisidiata*, *N.brasilisidiata*, *N.capensis*, *N.crassa*, *N.pauli*, *N.plurisporibadia* and *N.siamisidiata*. All *Neoprotoparmelia* species are well supported in the ML tree inferred from the ITS sequences (Fig. 1). The genus occurs throughout the tropics.

The presently available data do not allow us to infer the exact phylogenetic position of *Maronina* s.str. (*M.australiensis* and *M.hesperia*). The first 30 BLAST hits of the *M.australiensis* ITS fragment suggest close affinity of *M.australiensis* to *Lecanora* species.

### Distance summary

**Figure 2.**
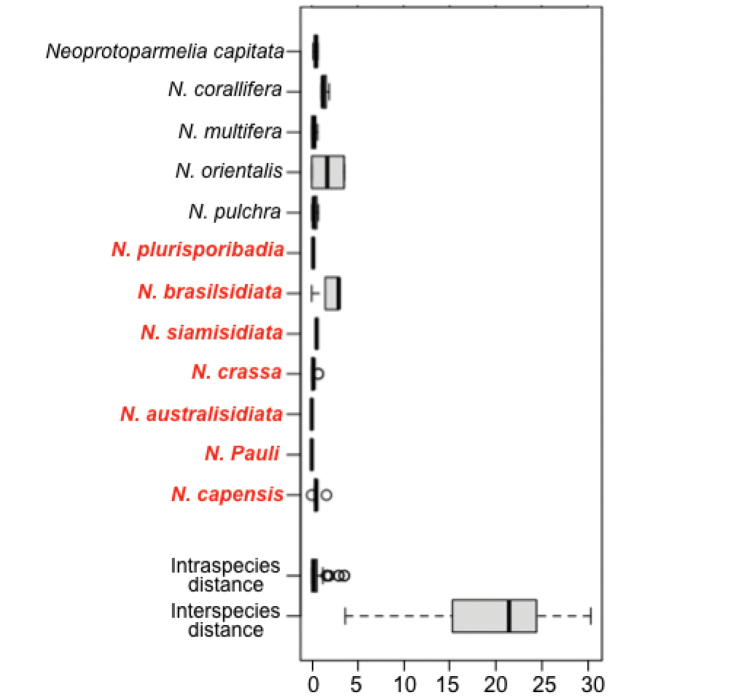
Boxplot showing the intra- and interspecific genetic distances of *Neoprotoparmelia* species and the overall intraspecific distances from all species and pairwise interspecific distances. New species described in this study are marked in red.

The mean intra- and inter-specific divergence was 0.56% (SE = 0.01) and 19.94 (SE = 0.01), respectively (Table 2). Our results thus show that, within species, divergence was much lower than inter-species divergence for all *Neoprotoparmelia* species (Table 2, Fig. 2). The maximum sequence divergence amongst individuals of a species was, in all cases, lower than the minimum interspecies sequence divergence, which supports the barcode-based taxonomic assignments of *Neoprotoparmelia* species (Table 2, Fig. 2). The maximum intraspecific genetic variation did not overlap with the nearest neighbour and a barcode gap was present amongst all neighbouring species (Fig. 2). Hence, we conclude that ITS is a suitable barcode marker to identify *Neoprotoparmelia* species.

**Table 2.** Genetic distances amongst *Neoprotoparmelia* species.

Species	Mean Intra-species distance	Max Intra-species distance	Nearest Neighbour	Distance to NN
The mean, maximum intra-specific distances and distance to the nearest neighbour				
* Neoprotoparmelia capitata *	0.43	0.69	* N. corallifera *	3.66
* N. corallifera *	1.39	1.92	* N. capitata *	3.66
* N. brasilisidiata *	1.97	2.95	* N. siamisidiata *	7.49
* N. siamisidiata *	0.54	0.54	* N. brasilisidiata *	7.49
* N. crassa *	0.23	0.71	* N. corallifera *	16.65
* N. australisidiata *	0.0	0.0	* N. corallifera *	18.45
* N. pauli *	0.0	0.0	* N. plurisporibadia *	13.09
* N. multifera *	0.25	0.64	* N. corallifera *	7.12
* N. orientalis *	1.75	3.57	* N. pulchra *	5.95
* N. pulchra *	0.32	0.72	* N. orientalis *	5.95
* N. capensis *	0.58	1.61	* N. plurisporibadia *	9.02
* N. plurisporibadia *	0.16	0.16	* N. capensis *	9.02
Intra-species and inter-species genetic distances				
Category	Minimum distance (%)	Mean distance (%)	Maximum distance (%)	SE distance
Intraspecific	0.00	0.56	3.57	0.01
Interspecific	3.66	19.94	30.34	0.01

## Taxonomic conclusions

### Maronina

Taxon classificationFungiLecanoralesParmeliaceae

Hafellner & R. W. Rogers, Biblioth. Lichenol. 38: 100. 1990

25517

Figure 3

#### Type species.

**Figure 3.**
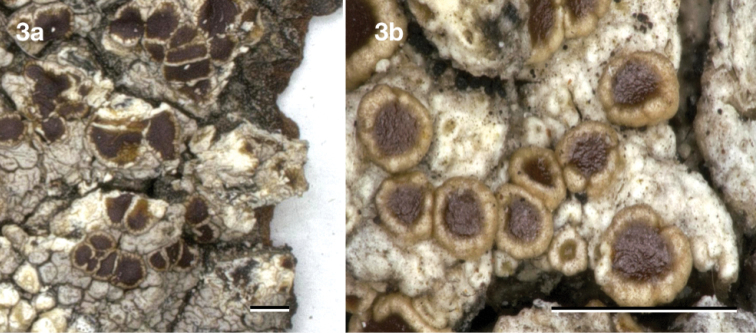
*Maroninaaustraliensis* (type species of *Maronina*), holotype Hafellner 17823 & Rogers (GZU). Scale bar: 1 mm.

*Maroninaaustraliensis* Hafellner & R. W. Rogers. Type. AUSTRALIA (Fig. 3). Queensland, Tandora about 25 km ENE of Maryborough, sea level, 25°27'S, 152°52'E, mangroves, on *Rhizophorastylosa*, 23 August 1986, J. Hafellner 17823 & R. W. Rogers (holotype GZU).

Based on molecular and phenotypical evidence, we propose *Maronina* s.str. to be a strictly Australian genus, comprising *M.australiensis* and *M.hesperia* Kantvilas & Elix only, as was suggested by Kantvilas and Elix (2007)) and Kantvilas et al. (2010). The genus *Maronina* contains depsides instead of depsidones as found in *Neoprotoparmelia*. Paraphyses in *Maronina* are slender and mostly simple, whereas those in *Neoprotoparmelia* are branched and anastomosing.

### Neoprotoparmelia

Taxon classificationFungiLecanoralesParmeliaceae

Garima Singh, Lumbsch & I. Schmitt gen. nov.

826940

Figures 4 , 5 , 6 , 7 , 8 , 9 , 10 , 11 , 12 , 13

#### Type species.

*Neoprotoparmeliacorallifera* (Kantvilas & Papong) Garima Singh, Lumbsch & I. Schmitt

#### Etymology.

Derived from the Greek *neos* (=new) and its close relationship to *Protoparmelia*.

#### Diagnosis.

Thallus crustose. Apothecia lecanorine, broadly adnate to sessile; thalline margin distinct. Proper excipulum cupulate, hyaline. Asci 8- to multispored, clavate, variations of the *Lecanora*-type (Hafellner 1984, Kantvilas and Elix 2007, Kantvilas et al. 2010). *Paraphyses* sparingly branched and anastomosing; apices clavate and brown-pigmented. Ascospores ellipsoid to fusiform to elongate, non-halonate. Pycnidia immersed, globose. Conidia bacilliform.

#### Chemistry.

*Neoprotoparmelia* species mainly produce depsidones of the alectoronic acid chemosyndrome.

#### Distribution and ecology.

The taxa of this genus occur in open habitats, mostly on bark, with only a few species growing on siliceous rock. This genus has a Pantropical distribution and is currently known from Australia, Brazil, Kenya, Papua New Guinea, South Africa, Thailand and south-eastern USA.

#### Remarks.

The new genus is morphologically similar to *Maronina* but can be distinguished by containing depsidones instead of depsides as found in *Maronina* and branched paraphyses. The genus is morphologically similar to *Protoparmelia* but was recognised as “tropical *Protoparmelia* clade” in Singh et al. (2015). The asci are essentially variations of the *Lecanora*-type sensu Hafellner (1984), and mainly coincides with those well studied by Kantvilas and Elix (2007) and Kantvilas et al. (2010). A detailed illustration of the ascus of *N.pulchra* is given in Aptroot et al. (1997a: 148, fig. 101a); it is similar to the ascus illustrated of *Protoparmeliabadia* by Hafellner (1984: 393, fig.40).

### Neoprotoparmelia amerisidiata

Taxon classificationFungiLecanoralesParmeliaceae

Garima Singh & Aptroot sp. nov.

827474

Figure 4

#### Type.

USA. Georgia, McIntosh Co., Sapelo Island, Sapelo Island Wildlife Management Area, 31°26'00"N, 81°22'10"W, on bark of *Quercus*, 16 December 2009, J. Lendemer 20995 (holotype: NY).

**Figure 4.**
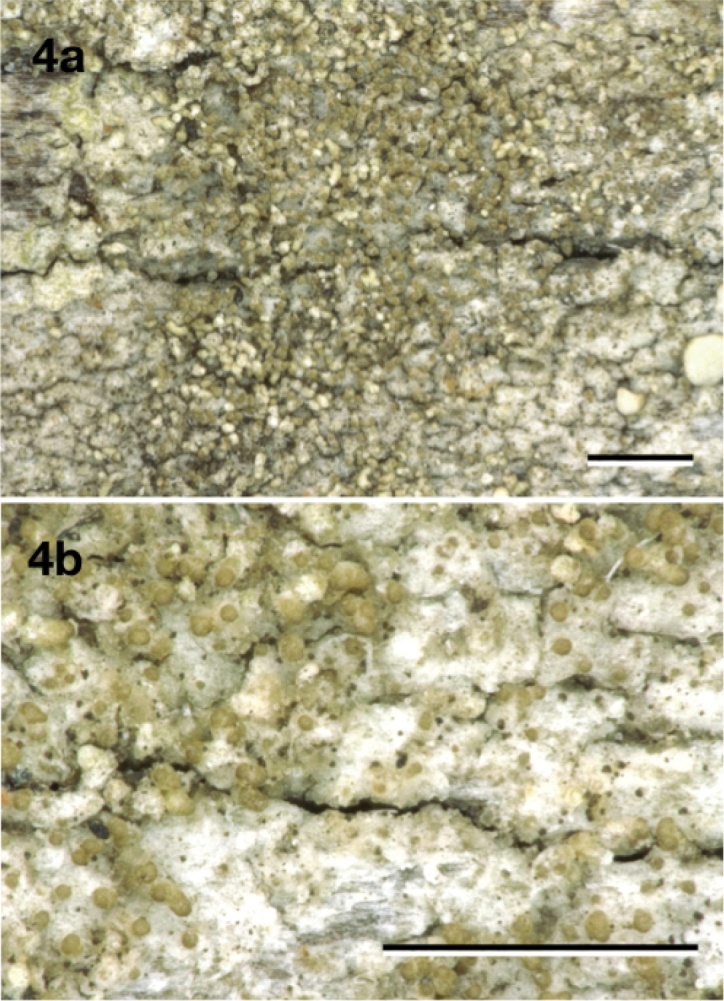
*Neoprotoparmeliaamerisidiata*, holotype Lendemer 20995 (NY). Scale bar: 1 mm.

#### Diagnosis.

Similar to *Neoprotoparmeliabrasilisidiata*, but differing by the thicker, 0.07–0.11 mm wide, isidia.

#### Etymology.

Named after its distribution in North America and the presence of isidia.

#### Description.

Thallus up to ca. 0.05 mm thick, shiny, pale olive-green to olive-grey, continuous, delimiting marginal prothallus line (brown, thin or absent). Isidia always numerous, initially widely dispersed or somewhat clustered, eventually covering much of the thallus, up to 1.5 mm long, persistently 0.07–0.11 mm wide over their whole length, cylindrical, usually irregularly repeatedly branched and somewhat nodulose, glossy, pale olive-green to olive-grey, tips distinctly brown and dull. Apothecia and pycnidia not observed.

#### Chemistry.

Spot tests: medulla of thallus and isidia UV++ greenish-white, C–, P–, K–, KC+ pink. TLC: alectoronic acid (major), dehydroalectoronic acid (minor or trace) and β-alectoronic acid (trace).

#### Distribution and ecology.

On tree bark in forest. Known only from the south-eastern USA (North Carolina, Alabama, Georgia, Mississippi and Florida).

#### Reference sequences.

(specimen: Lendemer 20995, holotype: NY). KY012827 (mtSSU), KY066301(nuLSU).

#### Remarks.

This species comprises the specimens recovered within ‘*P.isidiata* A’ in ‘*Protoparmelia* tropical clade’ in Singh et al. (2015). It is morphologically most similar to *N.brasilisidiata* which only differs by the generally thinner isidia. Some specimens have been reported before as *Protoparmeliaisidiata* (Lendemer and Lumbsch 2008).

#### Additional specimens examined.

USA. Florida, Gilchrist Co., Waccasassa Flats, 5 December 1993, R.C. Harris 31685, 31755 (NY), R.C. Harris 31685 (NY); USA. Georgia, McIntosh Co., Sapelo Island, Sapelo Island Wildlife Management Area, 15 December 2009, J. Lendemer 20745, 20727 (NY).

### Neoprotoparmelia australisidiata

Taxon classificationFungiLecanoralesParmeliaceae

Garima Singh & Aptroot sp. nov.

826943

Figure 5

#### Type.

AUSTRALIA. Northern Territory, 2 km N of Emerald Springs, 13°37'23"S, 131°36'40"E, on *Erythrophloeumchlorostachys*; 22 September 2007, G. Kantvilas 289/07 (holotype: HO 545660).

**Figure 5.**
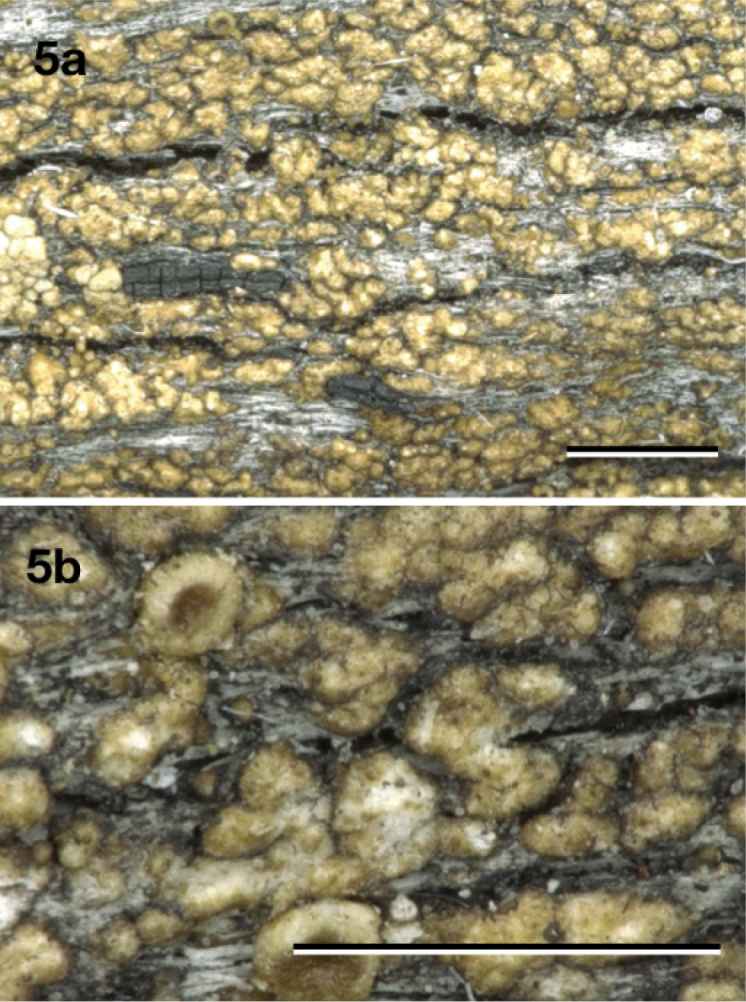
*Neoprotoparmeliaaustralisidiata*, holotype Kantvilas 289/07 (HO 545660). Scale bar: 1 mm.

#### Diagnosis.

Similar to *Neoprotoparmeliaisidiata*, but differing by the larger number of isidia per thallus areole.

#### Etymology.

Named after Australia and the presence of isidia.

#### Description.

Thallus consisting of almost contiguous, flat to convex areoles with irregular shape, of up to ca. 0.1 mm thick and 0.7 mm wide, somewhat shiny, pale brown to dark brown or pale olive-green to olive-grey, marginal prothallus black, thin or absent. Isidia usually in groups on almost each thallus areole, up to 0.9 mm long, persistently 0.07–0.1 mm wide over their whole length, cylindrical, usually rather irregularly once or more rarely repeatedly branched and somewhat nodulose, somewhat shiny, pale to dark brown or pale olive-green to olive-grey, of thallus colour, tips not darkened or somewhat brown. Apothecia (only young ones observed) sessile, round, 0.4–0.6 mm diam., disc concave to flat, smooth, glossy, orange brown. Margin glossy, ca. 0.05 mm wide, glossy brown at the outside, slightly higher than the disc. Hymenium hyaline, not inspersed with oil droplets, up to 50 μm high; epihymenium fuscous brown, pigment in K becoming soluble and paler; hypothecium hyaline, up to 90 μm thick including subhymenium; excipulum hyaline throughout, with a 5–12 μm thick layer of cortex, without crystals, with algae, extending below the hypothecium (cupulate). Paraphyses branched, ca. 2.5 μm wide, not thickened at the tips. Mature asci and ascospores not observed. Pycnidia not observed.

#### Chemistry.

Spot tests: medulla of thallus and isidia C–, P–, K–, KC+ pink, UV+ greenish-white. TLC: alectoronic acid (major), dehydroalectoronic acid (minor or trace) and β–alectoronic acid (trace).

#### Distribution and ecology.

On wood or bark of trees in open or closed forests. Known only from Australia (Northern Territory & New South Wales).

#### Reference sequences.

(specimen: Kantvilas 289/07, holotype: HO 545660). KP822276 (ITS), KP822466 (mtSSU), KP823523 (*TSR1*).

#### Remarks.

This species comprises the specimens recovered within ‘*P.isidiata* E’ in ‘*Protoparmelia* tropical clade’ in Singh et al. (2015) and referred to as *Maronina* in Divakar et al. (2017) and Singh et al. (2018). Coalescent-based species delimitation inferred from the six-locus dataset supports these taxa as distinct lineage from the other isidiate samples collected from the geographically distant populations. This species is morphologically very similar to *Neoprotoparmeliaisidiata*, but has larger and contiguous thallus areoles, usually bearing more isidia. Members of this species may differ considerably in colour and the abundance and maximum length of the isidia.

#### Additional specimens examined.

AUSTRALIA. New South Wales, Maxwells Flora Reserve, S of Eden, 195 m alt., 26 October 2010, G. Kantvilas 228/10 (HO 559228).

### Neoprotoparmelia brasilisidiata

Taxon classificationFungiLecanoralesParmeliaceae

Garima Singh, M. Cáceres & Aptroot sp. nov.

826944

Figure 6

#### Type.

BRAZIL. Sergipe, Parque Nacional Serra de Itabaiana, 10°44'57"S, 37°20'20"W, ca. 200 m alt., on bark of tree, 10 May 2014, M. Cáceres & A. Aptroot 21684 (holotype: ISE, isotype: ABL).

**Figure 6.**
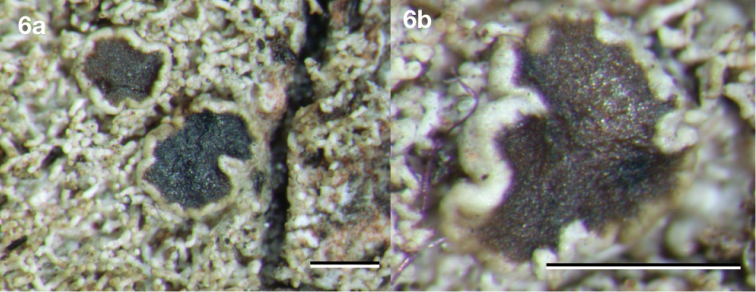
*Neoprotoparmeliabrasilisidiata*, holotype Cáceres & Aproot 21684 (ISE). Scale bar: 1 mm.

#### Diagnosis.

Very similar to *Neoprotoparmeliaamerisidiata*, but differing by having thinner, 0.04–0.08 mm wide, isidia.

#### Etymology.

Named after the country of discovery, Brazil and the presence of isidia.

#### Description.

Thallus up to ca. 0.05 mm thick, shiny, pale olive-green to olive-grey, continuous, marginal prothallus brown, thin or absent. Isidia always numerous, initially widely dispersed or somewhat clustered, eventually covering much of the thallus, up to 1.5 mm long, persistently 0.04–0.08 mm wide over their whole length, cylindrical, usually rather irregularly repeatedly branched and somewhat nodulose, glossy, pale olive-green to olive-grey, tips distinctly brown and dull. Apothecia sessile, round or usually with wavy outline, 0.6–1.3 mm diam., disc flat, smooth, dull, dark brown. Margin dull, ca. 0.15 mm wide, of thallus colour, not or only slightly higher than the disc. Hymenium hyaline, not inspersed with oil droplets, up to 80 μm high; epihymenium olive-brown, pigment in K becoming soluble and paler; hypothecium hyaline, up to 75 μm thick including subhymenium; excipulum hyaline throughout, with a 7–15 μm thick layer of cortex without crystals, with algae, extending below the hypothecium (cupulate). Paraphyses branched, ca. 2.0 μm wide, not thickened at the tips. Asci 8-spored, cylindrico-clavate, up to 55 × 13 μm. Ascospores hyaline, simple, narrowly ellipsoid, not constricted, 9–11 × 2–3 μm, without appendages. Pycnidia not observed.

#### Chemistry.

Spot tests: medulla of thallus and isidia UV+ greenish white, C–, P–, K–, KC+ pink. TLC: alectoronic acid (major), dehydroalectoronic acid (minor or trace) and β-alectoronic acid (trace). Gyrophoric acid has also been reported (Kalb 2004).

#### Distribution and ecology.

On tree bark in parks, open areas, Cerrado and Atlantic rain forests. Neotropical - known from Costa Rica, El Salvador and Brazil, where it is widespread and known from the following states: Sergipe, Matto Grosso, Rio de Janeiro, São Paulo, Maranhão, Tocantins, Minas Geraes and Rio Grande do Sul.

#### Reference sequences.

(specimen: Aptroot 21684, holotype: ISE). KY012831 (mtSSU), KY066305 (nuLSU).

#### Remarks.

This species comprises specimens recovered within ‘*P.isidiata* B’ in ‘*Protoparmelia* tropical clade’ in Singh et al. (2015). It is similar to *N.amerisidiata*, but, however, differs in having slightly thinner isidia. It is a common species on exposed bark in the neotropics and can easily be recognised in the field, from other isidiate crusts even when sterile, due to the strong UV-reaction visible with a portable UV-torch and thus can be distinguished from other isidiate crusts, even when sterile.

#### Additional specimens examined.

BRAZIL. Rio Grande do Sul, Viamão, near Parque Itapua, ca. 100 m alt.; 26 September 2014, M. Cáceres & A. Aptroot 22137 (ABL, ISE); Maranhão, Bananal, 20 km S of Imperatriz, ca. 140 m alt.; 20 October 2016, M. Cáceres & A. Aptroot 28776 (ABL, ISE). Tocantins, near Itaguatins, ca. 150 m alt.; 22 October 2016, M. Cáceres & A. Aptroot 28809 (ABL, ISE). COSTA RICA. Guanacaste, 15 km SSE of Nicoya, ca. 850 m alt.; 22 March 2004, H. Sipman 52086 (B), A. Aptroot 60835, 60836 & 60840 (INB). SAN SALVADOR. Ahuachapán, Parque Nacional El Imposible, ca. 800 m alt.; December 1998, R. Welz 89, 140 & 438 (B).

### Neoprotoparmelia capensis

Taxon classificationFungiLecanoralesParmeliaceae

V. J. Rico, A. Crespo & Garima Singh sp. nov.

826945

Figure 7

#### Type.

SOUTH AFRICA. Western Cape prov., between Papendorp and Strandfontein, near Vailkay bridge, 31°41'34"S, 18°13'59"E, ca. 32 m alt., 4 February 2005, A. Crespo, P.K. Divakar, D.L. Hawksworth, G. Amo & T.H. Lumbsch 63f (holotype: MAF–Lich. 19584; isotypes: MAF-Lich. 19624, 19625, 19626 and 19628).

**Figure 7.**
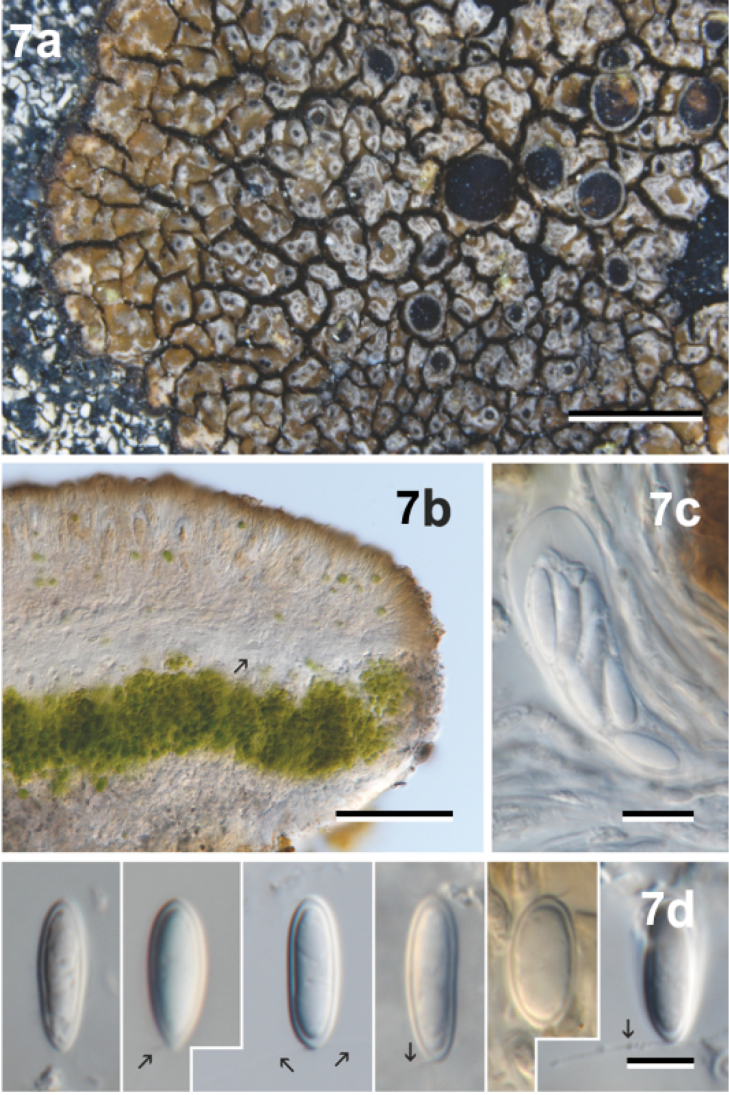
*Neoprotoparmeliacapensis*, holotype Crespo, Divakar, Hawksworth, Amo & Lumbsch 63f (MAF-Lich. 19584). **a** Habit **b** Section through centre of apothecium, showing cupular proper exciple (arrow) **c** Ascus **d** Spores, showing setae (arrow). Scale bars: 2 mm (**a**), 100 µm (**b**), 10 µm (**c**), 5 µm (**d**).

#### Diagnosis.

Morphologically similar to the northern hemispheric *Protoparmeliamontagnei* (Fr.) Poelt & Nimis, but mainly differing from it by the presence of alectoronic acid as major secondary metabolite in the medulla. The two species, *P.montagnei* and *N.capensis*, are also genetically not closely-related and belong to different genera.

#### Etymology.

The specific epithet refers to its occurrence in Cape Province of South Africa.

#### Description.

Thallus saxicolous, crustose, up to 8 cm wide, thin and areolate (in younger parts, up to 1 mm thick) to mainly thick and areolate, warted or subsquamulose (up to 2.2 mm thick), irregular or orbicular; surface light grey, pale to strong brown, with whitish mottled-fissured areas (by a locally strong mucilaginous epicortex), dull; delimited, or not, by a blackish hypothalline line. Areoles irregular, polygonal to rounded, up to 2 mm in diam., mainly slightly convex to irregular or flat, surface smooth to irregular, cracked or warted, marginal areoles sometimes lobe-like. Apothecia frequent, 1 to several per areolae, zeorine to lecanorine, immersed and nearly urceolate when young to adnate or sessile and constricted at the base when adult, rounded to irregular, up to 2 mm in diam.; disc brown to brown-black, dull, concave to flat or sometimes convex; thalline exciple persistent or excluded with age, concolorous with thallus to whitish (by a strong mucilaginous epicortex); proper exciple cupulate, up to 70–155 µm thick, coherent, hyphae mainly periclinal with strong mucilaginous walls, margins reduced in young apothecia. Hymenium hyaline to yellowish, coherent, 60–75 µm tall, in the margins somewhat fan-like (together with proper exciple) and exceeding the thalline exciple in adult apothecia; epihymenium light brown to brown, up to 15 µm tall, with few irregular granules; hypothecium and subhymenium hyaline to slightly yellowish, 25–70 µm thick. Paraphyses coherent in water, branched and anastomosed, apices somewhat thickened and mainly surrounded by a brown mucilaginous hood (up to 10 µm wide). Asci clavate, 42–70 × 12–20 µm, 8–spored, amyloid tholus (excluding the axial mass) and surrounding mucilage, *Lecanora*–type (cf. also *Maronina*–type, Kantvilas et al. 2010). Ascospores hyaline, simple, 9–13(–14) × 3.5–5.5(–6) µm (n = 40), fusiform to elongate (l:b = 1.8–2.9), with rounded apices or sometimes slightly apiculate in one end, some with apical hyaline setae. Pycnidia frequent, immersed, globose to oblong, wall hyaline, ostiole tissue with brown to black pigmented walls. Conidia simple, hyaline, 7–17 × 1–1.5 µm (n = 20), bacilliform, straight.

#### Chemistry.

Spot tests: medulla K– or ± unclean yellowish, C–, KC+ unclean rose-red, I–, P–, UV++ greenish-white. TLC: atranorin (traces), α–alectoronic acid (major), unidentified substance (major or traces, closed to norstictic acid, Rf class 4), ± β–alectoronic (traces) and traces of related substances.

#### Distribution and ecology.

Only known from the type locality in the arid north-west of the Cape Region (South Africa), rich in succulent plants (succulent Karoo biomes, cf. Mucina and Rutherford 2006), growing on exposed sandstones next to the Atlantic coast.

#### Reference sequences.

(specimen: Crespo, Divakar, Hawksworth, Amo & Lumbsch 63f, holotype: MAF–Lich. 19584). KY066279 (ITS), KP822500 (mtSSU), KP796385 (nuLSU), KP822184 (RPB1), KP823556 (TSR1).

#### Remarks.

This comprises the specimens recovered within ‘*P.* sp. ZA’ in ‘*Protoparmelia* tropical clade’ in Singh et al. (2015). *Neoprotoparmeliacapensis* is morphologically similar to the *Protoparmeliamontagnei* complex, in the sister genus *Protoparmelia*, but differs from the latter in its chemistry and distribution. The major secondary metabolite found in *N.capensis* is alectoronic acid whereas, in *P.montagnei*, it is lobaric and/or gyrophoric acids or fatty acids. *Protoparmeliamontagnei* is distributed in Eurasia on acid rocks, with mainly a broad Mediterranean distribution, from Turkey to The Canary Islands and from Ireland to Morocco (Coppins and Chambers 2009, Barbero et al. 2006). In contrast, *N.capensis* grows on sandstone in the Cape Region. Molecular data also clearly supports *N.capensis* and *P.montagnei* as distantly related, evolutionary independently lineages (Singh et al. 2015). Details on the morphology and chemistry of the similar *P.montagnei* species complex can be found in Coppins and Chambers (2009) and Barbero et al. (2006). The grey to brown thalli, 8-spored asci, α–collatolic acid absence, distribution and/or molecular data, supports *N.capensis* as an evolutionary independent lineage from the other two saxicolous *Neoprotoparmelia* species here described.

The analysed material of *Neoprotoparmeliacapensis* was rich in lichenicolous ascomycetes, some of which make its characterisation confusing. Portions of the studied specimens serve as host to species of *Phacographa* and *Sphaerellothecium* similar to those living on taxa of the *Protoparmeliabadia* complex (Hafellner 2009 and Triebel 1989, respectively), causing visible symptoms. A *Phoma*–type fungus, with hyaline pycnidia and conidia, frequently infected the hymenium of *N.capensis*. Moreover, in some adult apothecia of *N.capensis*, an endohymenial *Arthonia* species develops its asci, together with those of the host. The latter two taxa lacked visible symptoms on the host. These four lichenicolous fungi are currently under further investigation and the results will be published in a subsequent study.

### Neoprotoparmelia capitata

Taxon classificationFungiLecanoralesParmeliaceae

(Lendemer) Garima Singh, Lumbsch & I. Schmitt comb. nov.

827475

#### Basionym.

*Protoparmeliacapitata* Lendemer, Lichenologist 40: 332. 2008.

#### Synonym.

*Maroninacapitata* (Lendemer) Divakar, A. Crespo & Lumbsch in Divakar et al., Fungal Diversity 84: 114. 2017.

### Neoprotoparmelia corallifera

Taxon classificationFungiLecanoralesParmeliaceae

(Kantvilas & Papong) Garima Singh, Lumbsch & I. Schmitt comb. nov.

827476

Figure 8

#### Basionym.

Maroninaorientalisvar.corallifera Kantvilas & Papong in Kantvilas et al., Lichenologist 42: 557. 2010.

**Figure 8.**
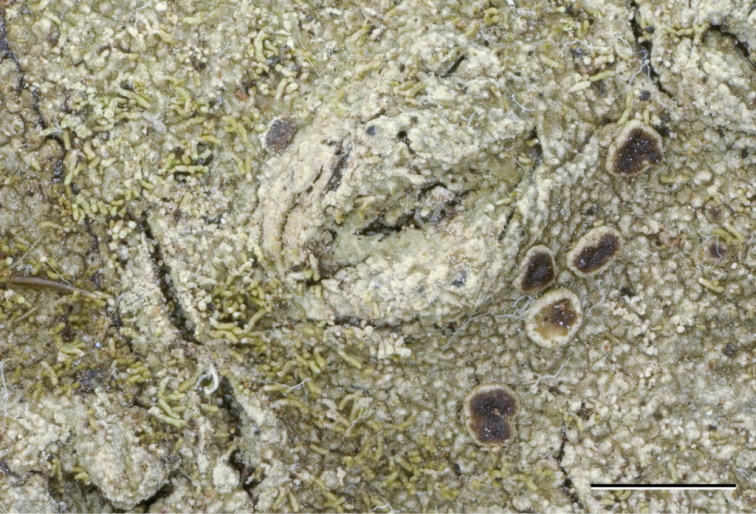
*Neoprotoparmeliacorallifera* (type species *Neoprotoparmelia*), sample Papong 7100. Scale bar: 1 mm.

#### Synonyms.

*Protoparmeliacorallifera* (Kantvilas & Papong) Kantvilas, Papong & Lumbsch in Papong et al., Lichenologist 43: 561–567. 2011. *Maroninacorallifera* (Kantvilas & Papong) Divakar, A. Crespo & Lumbsch in Divakar et al., Fungal Diversity 84: 114. 2017.

#### Type.

Thailand, Phu Pha Kham, Muk Dahan Province, Nhong Sung District, 16°46'N, 104°43'E, in dry dipterocarp forest, 310 m altitude, 21 June 2009, K. Papong & W. Konhin 6603 p.p.

### Neoprotoparmelia crassa

Taxon classificationFungiLecanoralesParmeliaceae

Garima Singh & Aptroot sp. nov.

827477

Figure 9

#### Type.

AUSTRALIA. Australian Capital Territory, Solar Village, J.A. Elix 39805 (holotype: CANB 783260).

**Figure 9.**
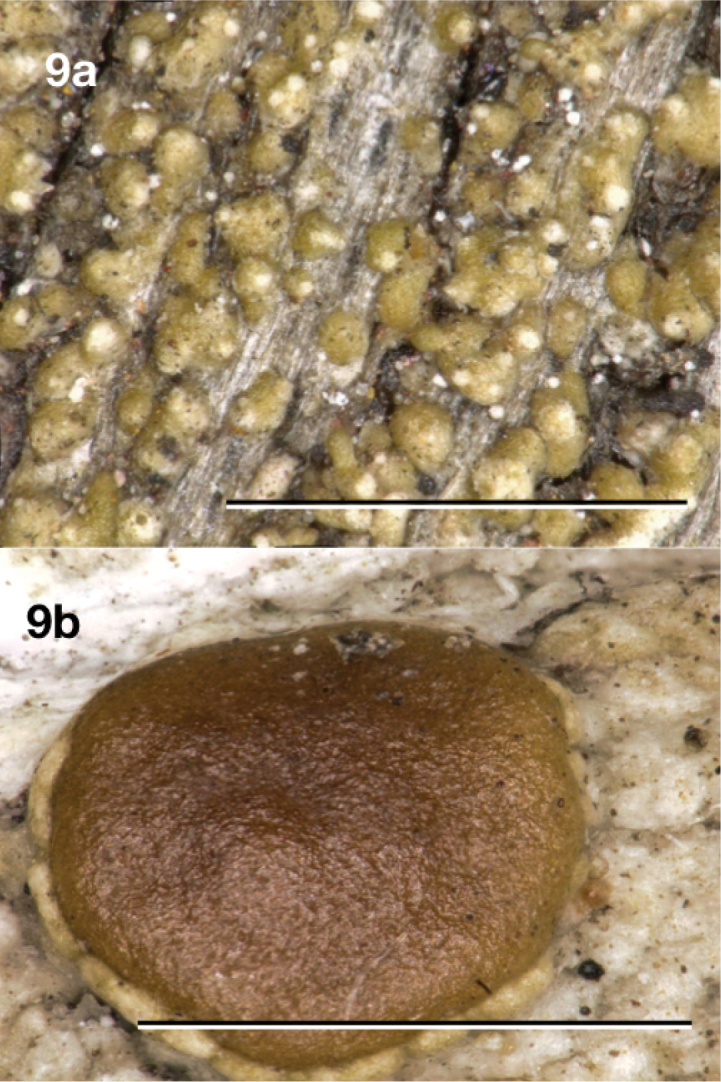
*Neoprotoparmeliacrassa* Elix39818. Scale bar: 1 mm.

#### Diagnosis.

Similar to *Neoprotoparmeliaisidiata*, but differs from it in having shorter isidia and a thicker thallus.

#### Etymology.

Derived from *crassus* (Lat. = fat) indicating that the thallus is thicker than that of the other isidiate species.

#### Description.

Thallus consisting of contiguous to centrally fusing, flat to rather convex areoles with irregular shape, of up to ca. 0.1 mm thick and 0.3 mm wide, somewhat shiny, pale brown to dark brown, marginal prothallus absent. Isidia covering most of the thallus except the outer margins, globose to ellipsoid, up to 0.15 mm long, persistently 0.07–0.1 mm wide, unbranched, of thallus colour, tips not darkened or somewhat brown. Apothecia and pycnidia not observed.

#### Chemistry.

Spot tests: medulla of thallus and isidia UV+ greenish white, C–, P–, K–, KC+ pink. TLC: alectoronic acid.

#### Distribution and ecology.

On wood or bark of trees in open or closed forests. Known only from Australia (Australian Capital Territory and Northern Territory).

#### Reference sequences.

(specimen: Elix 39805, holotype: CANB 783260). KP822464 (mtSSU), KP822274 (ITS), KP796345 (nuLSU), KP822145 (RPB1), KP822359 (MCM7), KP823521 (TSR1).

#### Remarks.

This comprises the specimens recovered within ‘*P.isidiata* D’ in ‘*Protoparmelia* tropical clade’ in Singh et al. (2015). Similar to *Neoprotoparmeliaisidiata* but differing in having a thicker thallus and shorter isidia.

#### Additional sequenced specimens examined.

AUSTRALIA. Same as type, J. A. Elix 39795 (CANB); Northern Territory, Melville Island, H. Streimann 42469 (B, CANB).

### Neoprotoparmelia isidiata

Taxon classificationFungiLecanoralesParmeliaceae

(Diederich, Aptroot & Sérus.) Garima Singh, Lumbsch & I. Schmitt comb. nov.

827478

Figure 10

#### Basionym.

*Protoparmeliaisidiata* Diederich, Aptroot & Sérus. in Aptroot et al., Biblioth. Lichenol. 64: 146. 1997.

**Figure 10.**
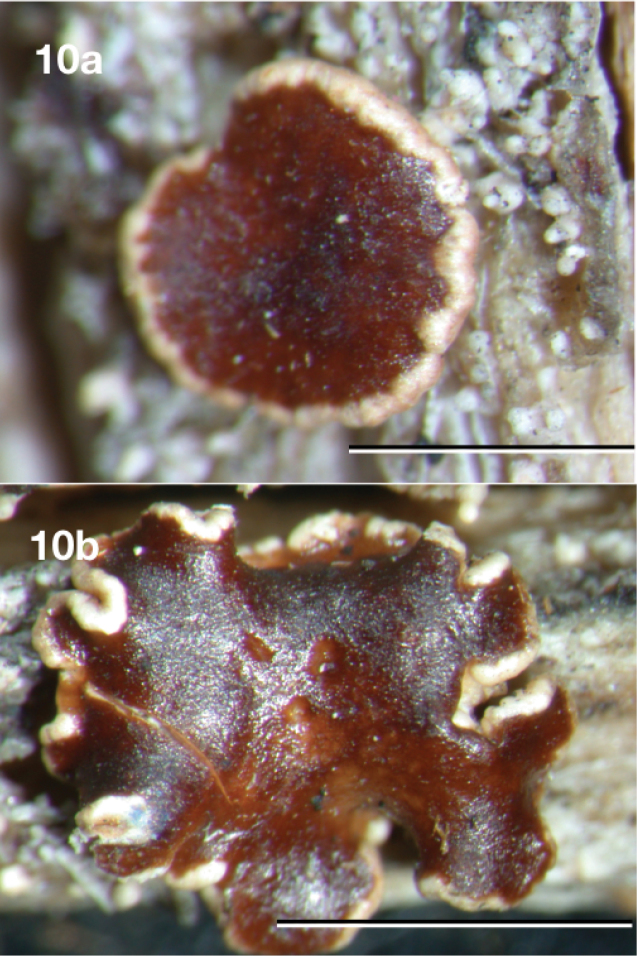
*Neoprotoparmeliaisidiata*, holotype Aptroot 31494 (BR). Scale bar: 1 mm.

#### Synonym.

*Maroninaisidiata* (Diederich, Aptroot & Sérus.) Divakar, A. Crespo & Lumbsch in Divakar et al., Fungal Diversity 84: 114 (2017).

#### Type.

PAPUA NEW GUINEA. Simbu, Mount Wilhelm, near lake Piunde, 5°47'S, 145°03'E, ca. 3600 m alt.; 5–8 August 1992, A. Aptroot 31494 (holotype: BR).

#### Description.

Thallus consisting of isolated convex areoles of up to ca. 0.1 mm thick and 0.2 mm wide, somewhat shiny, pale brown to dark brown or mottled whitish-grey, on a fully immersed hyaline hypothallus, marginal prothallus black, thin or absent. Isidia usually solitary on almost each thallus areole, up to 0.5 mm long, persistently 0.07–0.1 mm wide over their whole length, cylindrical, usually rather irregularly once or more rarely repeatedly branched and somewhat nodulose, glossy, pale to dark brown, tips dark brown to almost black. Apothecia sessile, initially round, older ones usually with wavy outline, 0.6–3.5 mm diam., disc flat, smooth, glossy, dark brown to orange brown. Margin glossy, ca. 0.25 mm wide, glossy brown at the outside, not or only slightly higher than the disc. Hymenium hyaline, not inspersed with oil droplets, up to 70 μm high; epihymenium fuscous brown, pigment in K becoming soluble and paler; hypothecium hyaline, up to 120 μm thick including subhymenium; excipulum hyaline throughout, with a 20–30 μm thick layer of cortex, without crystals, with algae, extending below the hypothecium (cupulate). Paraphyses branched, ca. 2.5 μm wide, not thickened at the tips. Asci cylindrico-clavate, up to 35 × 9 μm, with 8 mostly biseriate ascospores. Ascospores hyaline, simple, narrowly ellipsoid, not constricted, (9–)11–13(–17) × 2–3 μm, without appendages. Pycnidia not observed.

#### Chemistry.

Spot tests: medulla of thallus and isidia UV++ greenish-white, C–, P–, K–, KC+ pink. TLC: alectoronic acid (major), dehydroalectoronic acid (minor or trace) and β-alectoronic acid (trace).

#### Distribution and ecology.

On bark of trees in forests. Known from Papua New Guinea only.

#### Remarks.

This species differs from the other species by having a thallus consisting of tiny areoles, generally bearing just one isidium each and by large apothecia.

#### Additional specimens examined.

PAPUA NEW GUINEA. Simbu, Mount Wilhelm, near lake Piunde, ca. 3600 m alt.; 5–8 August 1992, A. Aptroot 32711 (BR); P. Diederich 10359 (Hb. Diederich); March 1987, A. Aptroot 18353 (BR).

### Neoprotoparmelia multifera

Taxon classificationFungiLecanoralesParmeliaceae

(Nyl.) Garima Singh, Lumbsch & I. Schmitt comb. nov.

827479

#### Basionym.

*Lecanoramultifera* Nyl., Acta Soc. Sci. Fenn. 7: 445. 1863.

#### Synonyms.

*Maroneamultifera* (Nyl.) Vain., Acta Soc. Fauna Flora Fenn. 7: 100. 1890. *Maroninamultifera* (Nyl.) Hafellner & R.W. Rogers, Biblioth. Lichenol. 38: 106. 1990. *Protoparmeliamultifera* (Nyl.) Kantvilas, Papong & Lumbsch in Papong et al., Lichenologist 43: 566. 2011.

### Neoprotoparmelia orientalis

Taxon classificationFungiLecanoralesParmeliaceae

(Kantvilas & Papong) Garima Singh, Lumbsch & I. Schmitt comb. nov.

827480

#### Basionym.

*Maroninaorientalis* Kantvilas & Papong in Kantvilas et al., Lichenologist 42: 557. 2010.

#### Synonym.

*Protoparmeliaorientalis* (Kantvilas & Papong) Kantvilas, Papong & Lumbsch in Papong et al., Lichenologist 43: 566. 2011.

### Neoprotoparmelia pauli

Taxon classificationFungiLecanoralesParmeliaceae

V. J. Rico, Lumbsch & Garima Singh sp. nov.

827481

Figure 11

#### Type.

KENYA. Eastern Prov., Mwingi Co., Nuu Hill, 01°02'S, 38°20'E, ca. 1000 m alt., inselberg with dry woodland dominated by *Terminalia*, *Combretum* and *Acacia*, on sandstone, 12 March 2014, P.M. Kirika & H.T. Lumbsch 3821 (holotype: EA, isotype: F).

**Figure 11.**
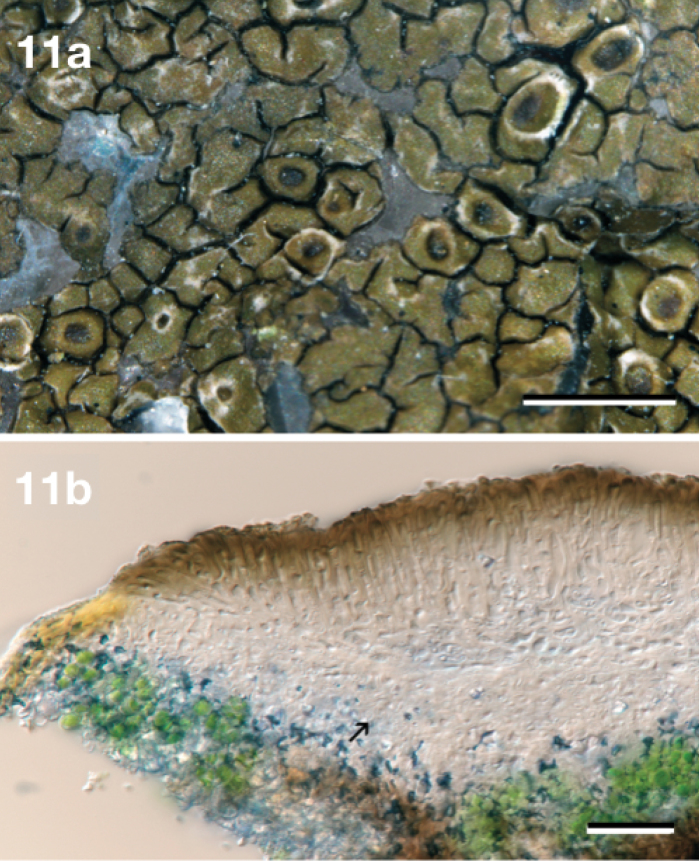
*Neoprotoparmeliapauli*, holotype Kirika & Lumbsch 3821 (EA) **a** Habit **b** Centre of apothecia section, showing cupular proper exciple (arrow). Scale bars: 1 mm (**a**), 20 µm (**b**).

#### Diagnosis.

Similar to *Neoprotoparmeliacapensis* but differs from it by having a reduced, olive tinged thallus and smaller apothecia. Moreover, the major secondary metabolite produced by *Neoprotoparmeliapauli* is α–collatolic acid, absent in *N.capensis*.

#### Etymology.

The new species is named after our colleague, the Kenyan lichenologist, Paul M. Kirika, who was one of the collectors of the type material.

#### Description.

Thallus saxicolous, crustose, up to 3 cm wide, rimose to areolate, thin (up to 0.8 mm thick); surface dark brown, olive-brown to light olive-brown, sometimes with whitish mottled-fissured areas (by a locally strong mucilaginous epicortex), dull to slightly shiny; blackish hypothalline line blackish or absent. Areoles irregular, polygonal to rounded, up to 0.75(–1.2) mm in diam., flat to slightly convex, surface mainly smooth, marginal areoles sometimes lobe-like. Apothecia frequent, 1 per areolae, zeorine to lecanorine, mainly immersed and nearly urceolate or adnate, rounded, up to 0.4 mm in diam.; disc brown to brown-black, dull, concave to flat; thalline exciple persistent, concolorous with thallus to whitish (by a strong mucilaginous epicortex); proper exciple cupulate, up to 35 µm thick, coherent, hyphae mainly periclinal with strong mucilaginous walls. Hymenium hyaline, coherent, 35–60 µm tall; epihymenium light brown to brown, up to 15 µm tall, with few irregular granules; hypothecium and subhymenium hyaline, 15–35 µm thick. Paraphyses coherent in water, branched and anastomosed, apices somewhat thickened and mainly surrounded by a brown mucilaginous hood (up to 7.5 µm wide). Asci clavate, 50 ×16 µm, 8–spored, amyloid tholus (excluding the axial mass) and surrounding mucilage, *Lecanora*–type (cf. also *Maronina*–type, Kantvilas et al. 2010). Ascospores hyaline, simple, 10–12.5 × 4–5 µm (n = 8), fusiform to elongate (l:b = 2–2.75), with rounded apices or sometimes slightly apiculate in one end, some with apical hyaline setae. Pycnidia immersed, globose to oblong, wall hyaline, ostiole tissue with brown pigmented walls. Conidia simple, hyaline, (9–)10–17 × 1–1.5 µm (n = 20), bacilliform, straight.

#### Chemistry.

Spot tests: medulla K– or ± unclean yellowish, C–, KC–, I–, P–, UV+ greenish-white. TLC: atranorin (minor or traces), α–collatolic acid (major or minor), α–alectoronic acid (minor), unidentified substance (major or traces, closed to norstictic acid, Rf class 4), ± β–alectoronic (traces) and traces of related substances.

#### Distribution and ecology.

Only known from the type locality in Kenya, covered with upland dry forest ecosystems (Wass 1995), growing on exposed sandstones.

#### Reference sequences.

(specimen: Kirika & Lumbsch 3821, holotype: EA). KP822469 (mtSSU), KP822279 (ITS), KP796348 (nuLSU), KP822148 (RPB1), KP823526 (TSR1).

#### Remarks.

Consists of specimens recovered within ‘*P.* sp. KE’ in ‘*Protoparmelia* tropical clade’ in Singh et al. (2015), supported as an evolutionary independent lineage based on the coalescent-based species delimitation analysis. The thalli of the type material were poorly developed, immature apothecia and only a few mature spores were found. This hindered us in providing detailed morphological features (especially ascomatal) and thus future collections may slightly change the morphological description. Its olive-brown thalli, 8-spored asci, α–collatolic acid presence, distribution and/or molecular data supports it as an evolutionary independent lineage from the other two saxicolous *Neoprotoparmelia* species.

### Neoprotoparmelia plurisporibadia

Taxon classificationFungiLecanoralesParmeliaceae

Garima Singh, M. Cáceres & Aptroot sp. nov.

827482

Figure 12

#### Type.

BRAZIL. Rio Grande do Sul, Viamão, near Parque Itapua, 30°05'S, 51°00'W, on granite, ca. 100 m alt.; 26 September 2014, M. Cáceres & A. Aptroot 22130 (holotype: ABL; isotype: ISE).

**Figure 12.**
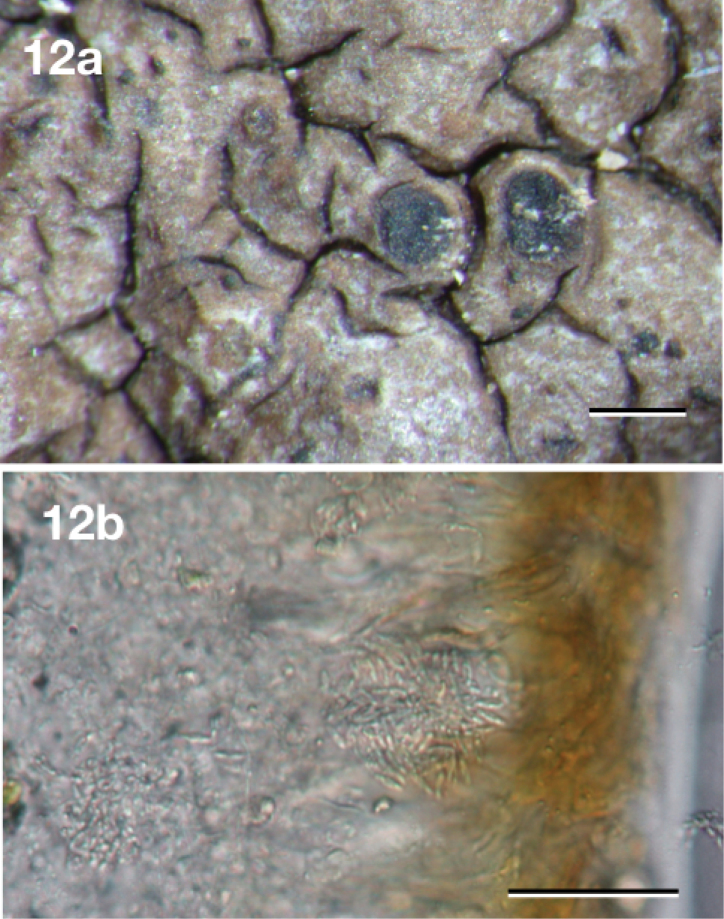
*Neoprotoparmeliaplurisporibadia*, holotype Cáceres & Aproot 22130 (ABL). **a** Habitus **b** ascus with ascospores. Scale bars: 1 mm (**a**), 10 micron (**b**).

#### Diagnosis.

Differing from the morphologically similar *Protoparmeliabadia* (Ach.) M. Choisy by the presence of multispored asci and different chemistry and distribution.

#### Etymology.

Named after *pluri* = many, spores and *badia* = dark brown.

#### Description.

Thallus consisting of areoles with wavy border of up to ca. 1.3 mm thick and 2.0 mm wide (but mostly much smaller) that are tightly packed together and occasionally become almost lobe-like, somewhat shiny, pale brown to dark brown, marginal prothallus black, thin or absent. Isidia absent. Apothecia immersed in areoles to erumpent, usually up to one per areole, initially round, later usually compressed and with wavy elongated shape, 0.4–1.3 mm diam., disc concave to flat, smooth, glossy, dark brown. Margin dull, ca. 0.3 mm wide, indistinguishable from the thallus, not or only slightly higher than the disc. Hymenium hyaline, not inspersed with oil droplets, up to 100 μm high; epihymenium fuscous brown, pigment in KOH becoming soluble and paler; hypothecium hyaline, not distinguishable from the thallus medulla and thus extending to over 1 mm; excipulum hyaline throughout, with a 10–15 μm thick layer of pseudocortex without crystals, with algae, not extending below the hypothecium. Paraphyses simple to somewhat branched, ca. 2.5 μm wide, not thickened at the tips. Asci cylindrico-clavate, blue, up to 95 × 15 μm, with ca. 50 ascospores. Ascospores hyaline, simple or occasionally with a pseudoseptum, narrowly ellipsoid, not constricted, 7.0–8.0 × 2.5–3.5 μm, wall ca. 0.5 μm thick, without appendages. Pycnidia abundant, immersed, dark brown; surrounding areole usually slightly raised. Conidia hyaline, linear to slightly clavate, 5–7.5 × 0.9–1.1 μm.

#### Chemistry.

Spot tests: medulla of thallus UV+ greenish-white, C–, P–, K–, KC+ pink. TLC: alectoronic acid.

#### Distribution and ecology.

On granite in open low mountain area. Known only from Brazil (Rio Grande do Sul).

#### Reference sequences.

M. Cáceres & A. Aptroot 22130, MK046748.

#### Remarks.

Somewhat similar to *Protoparmeliabadia*, from which it differs markedly by the multispored ascus and production of alectoronic acid instead of lobaric acid, as occurs in *P.badia*. It can also be distinguished from the other two saxicolous *Neoprotoparmelia* species, *N.pauli*, and *N.capensis*, by distribution and by the presence of approximately 50-spored asci in contrast to the 8-spored asci present in the latter.

### Neoprotoparmelia pulchra

Taxon classificationFungiLecanoralesParmeliaceae

(Diederich, Aptroot & Sérus.) Garima Singh, Lumbsch & I. Schmitt comb. nov.

827483

#### Basionym.

*Protoparmeliapulchra* Diederich, Aptroot & Sérus. in Aptroot et al., Biblioth. Lichenol. 64: 147. 1997.

TYPE: on the S shore of L. Piunde, Pindaunde Valley, Mt Wilhelm, Simbu Province, Papua New Guinea, 05°47'S, 145°43'E, alt. 3600 m, subalpine forest remnants on W slope of valley, 6 Aug. 1992, H. Sipman 35638; holo: B.

#### Synonym.

*Maroninapulchra* (Diederich, Aptroot & Sérus.) Divakar, A. Crespo & Lumbsch in Divakar et al., Fungal Diversity 84: 114. 2017.

### Neoprotoparmelia siamisidiata

Taxon classificationFungiLecanoralesParmeliaceae

Garima Singh & Aptroot sp. nov.

827684

Figure 13

#### Type.

THAILAND. Chiang Mai, Doi Suthep–Ou National Park, Medicinal Garden 18°48'17"N, 98°54'43"E, ca. 1100 m alt., on bark of *Cinchonapubescens*, 13 October 2002, H.J.M. Sipman 48520 (holotype: B).

**Figure 13.**
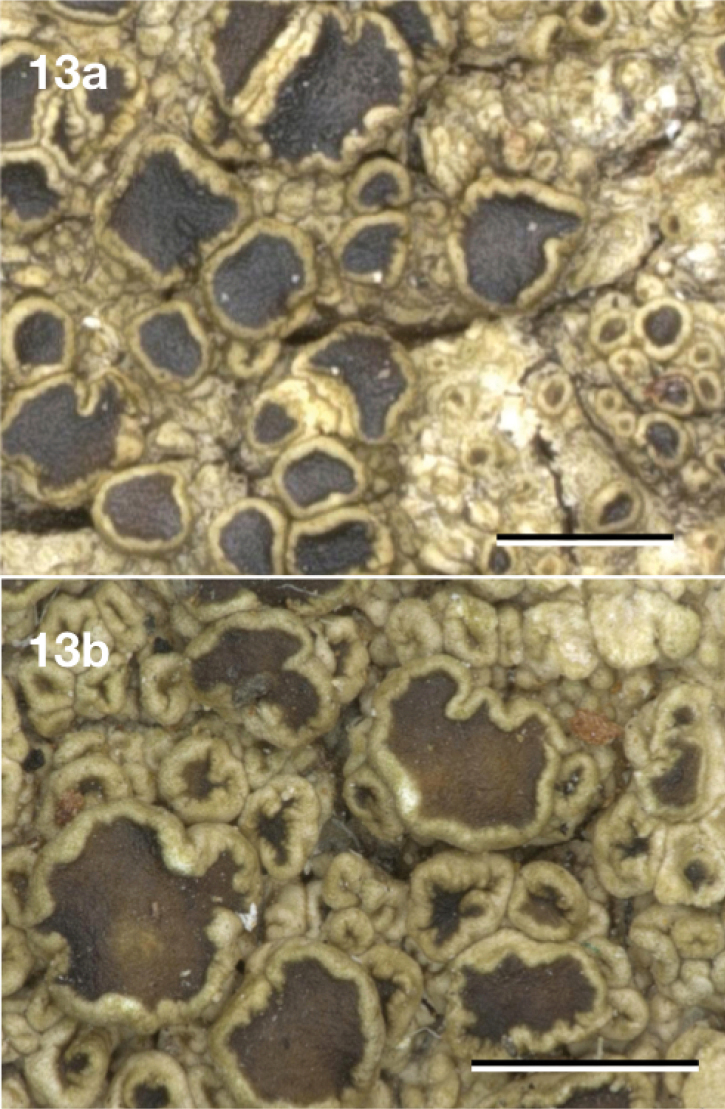
*Neoprotoparmeliasiamisidiata*, *v.d.* Boom 46872 (Hb. v.d. Boom). Scale bar: 1 mm.

#### Diagnosis.

Similar to *Neoprotoparmeliabrasilisidiata*, but mainly differs from it by the presence of 16–spored asci.

#### Etymology.

Named after the place of discovery, Siam (Thailand) and the presence of isidia.

#### Description.

Thallus consisting of slightly convex areoles of up to ca. 0.1 mm thick and 0.3 mm wide which are mostly coalescent to form a rimose thallus, somewhat shiny, pale brown to dark brown or mottled whitish-grey, on a fully immersed dark hypothallus, marginal prothallus black, thin or absent. Isidia always numerous, initially widely dispersed or somewhat clustered, eventually covering much of the thallus, up to 1.5 mm long, persistently 0.05–0.07 mm wide over their whole length, cylindrical, usually rather irregularly once or repeatedly branched and somewhat nodulose, glossy, pale to dark brown, tips generally dark brown. Apothecia sessile, initially round, older ones usually with wavy boundaries, 0.6–1.5 mm diam., disc flat, smooth, glossy, dark brown to orange brown. Margin glossy, ca. 0.25 mm wide, glossy brown at the outside, not or only slightly higher than the disc. Hymenium hyaline, not inspersed with oil droplets, up to 90 μm high; epihymenium fuscous brown, pigment in KOH becoming soluble and paler; hypothecium hyaline, up to 120 μm thick including subhymenium; excipulum hyaline throughout, with a 20–30 μm thick layer of cortex, without crystals, with algae, extending below the hypothecium (cupulate). Paraphyses branched, ca. 2.5 μm wide, not thickened at the tips. Asci cylindrico-clavate, blue, up to 35 × 9 μm, with 16 mostly biseriate ascospores. Ascospores hyaline, simple, broadly ellipsoid, not constricted, 9–11 × 6.5–8 μm, without appendages. Pycnidia not observed.

#### Chemistry.

Spot tests: medulla of thallus and isidia UV+ greenish-white, C–, P–, K–, KC+ pink. TLC: alectoronic acid (major), dehydroalectoronic acid (minor or trace) and β-alectoronic acid (trace).

#### Distribution and ecology.

On tree bark in a Park. Known only from Thailand (Chiang Mai).

#### Remarks.

This comprises the specimens recovered within ‘*P.isidiata* C’ in ‘*Protoparmelia* tropical clade’ in Singh et al. (2015). It is similar to the other four isidiate *Neoprotoparmelia* species but can be distinguished from them by the presence of 16-spored asci. For additional specimens from Thailand, see Aptroot et al. (2007, as *Protoparmeliaisidiata*). It can be distinguished from *Neoprotoparmeliacorallifera* only by presence of 8-spored asci (Aproot et al. 1997a) and by using molecular data.

### Key to *Neoprotoparmelia*

**Table:** 

1	Thallus sorediate or isidiate	**2**
–	Thallus lacking soredia or isidia	**9**
2	Thallus sorediate, known from USA and Brazil	*** Neoprotoparmelia capitata ***
–	Thallus isidiate	**3**
3	Isidia globose to ellipsoid, covering the thallus except margins, Australia	*** N. crassa ***
–	Isidia otherwise	**4**
4	Isidia up to 1.5 mm tall	**5**
–	Isidia less than 1.5 mm tall	**7**
5	Asci 16-spored, Thailand	*** N. siamisidiata ***
–	Asci 8-spored	**6**
6	Isidia persistently 0.07–0.11 mm wide over their whole length, SE of the USA	*** N. amerisidiata ***
–	Isidia thinner and less regular, South and Central tropical America	*** N. brasilisidiata ***
7	Asci 32–50-spored, Thailand	*** N. corallifera ***
–	Asci 8-spored, Australia or Papua New Guinea	**8**
8	Usually several isidia on one thallus areole, Australia	*** N. australisidiata ***
–	Each thallus areole with only one isidium, Papua New Guinea	*** N. isidiata ***
9	Thallus epiphytic	**10**
–	Thallus saxicolous	**12**
10	Asci 8-spored, Papua New Guinea	*** N. pulchra ***
–	Asci multispored	**11**
11	Asci 32-spored, South America	*** N. multifera ***
–	Asci 32–50-spored, Thailand	*** N. orientalis ***
12	Asci multispored, Brazil	*** N. plurisporibadia ***
–	Asci 8-spored	**13**
13	Thallus grey to brown, main substance alectoronic acid, South Africa	*** N. capensis ***
–	Thallus olive, main substance α-collatolic acid, Kenya	*** N. pauli ***

## Supplementary Material

XML Treatment for Maronina

XML Treatment for Neoprotoparmelia

XML Treatment for Neoprotoparmelia amerisidiata

XML Treatment for Neoprotoparmelia australisidiata

XML Treatment for Neoprotoparmelia brasilisidiata

XML Treatment for Neoprotoparmelia capensis

XML Treatment for Neoprotoparmelia capitata

XML Treatment for Neoprotoparmelia corallifera

XML Treatment for Neoprotoparmelia crassa

XML Treatment for Neoprotoparmelia isidiata

XML Treatment for Neoprotoparmelia multifera

XML Treatment for Neoprotoparmelia orientalis

XML Treatment for Neoprotoparmelia pauli

XML Treatment for Neoprotoparmelia plurisporibadia

XML Treatment for Neoprotoparmelia pulchra

XML Treatment for Neoprotoparmelia siamisidiata

## Supplementary materials

Supplementary material 1 Table S1: List of primers used in this study Data type: molecular data This is an open access article distributed under the terms of the Creative Commons Attribution License (CC BY 4.0), which permits unrestricted use, distribution, and reproduction in any medium, provided the original author and source are credited.Garima Singh, André Aptroot, Víctor J. Rico, Jürgen Otte, Pradeep K. Divakar, Ana Crespo, Marcela Eugenia da Silva Cáceres, H. Thorsten Lumbsch, Imke Schmitt
